# San Marino

**Published:** 1889-03

**Authors:** 


### SAN MARINO.

There are in Europe several states, not larger than ordinary townships in England or America, yet permitted to enjoy perfect independence of the powerful governments which surround them. That which has least escaped notice is the ancient republic of San Marino, so called, as some say after an old monk who was its founder though another account is that this honor is due to a pious mason of Delmatia in the fourth century. It is a craggy tract of country in the hills near Rimini, on the Adriatic, and includes about 33 square miles, with a population of about 8,000 and an army of 40 men, commanded by several "generals," for it is said, that offices and titles and decorations are to be had for a suitable consideration, independent of meritorious service. Though enclosed on all sides by what were the dominions of the Pope, and though it was severely menaced by Napoleon I in his conquering career, yet he was induced to respect its venerable autocracy and so have all the powers into whose hands the surrounding parts of Italy have fallen both before and since ; so that this little republic has continued independent for at least fourteen centuries, a longer period than any other government in Europe can boast of. Austria, Prussia and France have each had 1,100 years of united independence, England 800, and Russia 350 years only. It has an unwritten constitution, according to which the legislative power is vested in a council of 60, elected by the people. Of these twenty are nobles ; 20 are townspeople ; and 20 are from the rural population. The executive lies with two of the councillors, who are chosen every six months and act jointly as regents. The judicial power is exercised by a doctor of laws, who must be a stranger, and cannot hold office longer than three years. The village of 1,500 people, which forms the capital of the republic, is situated high up on Mount Titan. It has a castle, which was fortified by King Berenger, of Lombardy, and as its principal object of interest, a splendid collection of medals numbering about 40,000. The principal inhabitants reside in a more sheltered locality. The people generally are in a very backward condition. They have no printing press but rejoice in four convents, five churches and a theatre.

